# Correlates of Insufficient Physical Activity among Junior High School Students: A Cross-Sectional Study in Xi’an, China

**DOI:** 10.3390/ijerph13040397

**Published:** 2016-04-01

**Authors:** Xiaoqin Wang, Zhaozhao Hui, Paul D. Terry, Mei Ma, Li Cheng, Fu Deng, Wei Gu, Bin Zhang

**Affiliations:** 1 School of Medicine, Xi’an Jiaotong University, Xi’an 710061, Shaanxi Province, China; huizhaozhao93@163.com (Z.H.); wysun201314195@163.com (M.M.); 232guwei@mail.xjtu.edu.cn (W.G.); zhbin@mail.xjtu.edu.cn (B.Z.); 2 Department of Medicine, University of Tennessee Medical Center, Knoxville, TN 37996, USA; pdterry@utk.edu; 3 Faculty of Medicine, The Chinese University of Hong Kong, Shatin 999077, Hong Kong, China; lilycheng@link.cuhk.edu.hk; 4 Xi’an Tie Yi High School, Xi’an 710000, Shaanxi Province, China; dengfu01@126.com

**Keywords:** MVPA, adolescent, obesity, socioeconomic status (SES), family support

## Abstract

*Background*: Physical activity plays an important role in individual health at all stages of life. However, evidence is lacking regarding the level of moderate to vigorous physical activity (MVPA) and the related factors to insufficient physical activity (IPA). *Methods*: A sample of 1060 students aged 12–15 years from nine public junior high schools in China were invited to participate in this study. Physical activity was assessed by a modified version of the International Physical Activity Questionnaire. Logistic regression was used to assess the factors associated with IPA. *Results*: 30.1% boys and 43.0% girls in our sample did not engage in sufficient physical activity. Obesity (OR = 2.2, 95% CI: 1.5–2.9) and high socioeconomic status (SES) (OR = 2.4, 95% CI: 1.3–4.8) were positively associated with IPA, whereas male sex (OR = 0.7, 95% CI: 0.5–0.9), underweight status (OR = 0.5, 95% CI: 0.3–0.7), and high family support level for physical activity (OR = 0.7, 95% CI: 0.6–0.9) showed inverse associations. Age was not associated with IPA. *Conclusions*: IPA appears to be a considerable problem in this sample of Chinese youth. Effective interventions to increase physical activity are needed and may include improving family support level for physical activity, especially for girls, the obese and those with high SES.

## 1. Introduction

The World Health Organization (WHO) recommends that children and adolescents aged 5–17 years old should accumulate at least 60 minutes of moderate to vigorous physical activity (MVPA) daily [1]. Physical activity below this level is considered insufficient. A recent systemic review [2] showed that the worldwide prevalence of insufficient physical activity (IPA) varied from 18.7% to 90.6%, with a median of 79.7%. The U.S. National Survey of Children’s Health found that only 28% of U.S. children aged 6–17 participated in daily vigorous physical activity in 2011–2012 [3]. Decelis *et al.* found that only 39% of boys and 10% of girls met the recommendation of one hour of MVPA in Malta [4]. In Taiwan, it was found that 40.9% of the adolescent girls and 24.0% of the adolescent boys did not achieve the WHO recommendations for physical activity [5]. A cross-sectional study in Beijing showed that 30.7%–61.3% of middle school students did not meet the current physical activity recommendations, and about half of the students spent excessive time engaging in sedentary behaviors [6]. In these studies, it is possible that measurement error led to underestimates or overestimates of physical activity to varying degrees. Nonetheless, given the consistency of findings across countries, there appears to be a global pandemic of IPA.

IPA has a major influence on the prevalence of chronic and non-communicable diseases such as cardiovascular disease, obesity, diabetes, hypertension, and cancer [7,8,9]. For adolescents, IPA has also been shown to have deleterious effects on body weight and body composition [10], which has social and psychological implications [11,12]. Indeed, IPA has been linked with increased risk of mental health problems [11], whereas it has been noted that adequate physical activity may reduce symptoms of depression [12]. In addition, a study based on 2225 students in Shanghai suggested that physical activity was associated with higher scholastic performance [13]. Although there are many possible reasons for this latter association, research has demonstrated improvements in cognitive performance in individuals who were physically active during childhood and adolescence [14].

In the past decade, epidemiological studies have examined factors associated with IPA, including gender [5,6], age [15,16], social support for physical activity [17,18], body weight [19], and socioeconomic status (SES) [20]. Parents may often exert considerable influence on the physical activity-related behaviors of youth, with either tangible or intangible support. For instance, parents provide transportation to places where the children can engage in a variety of physical activities, purchase equipment, play with their children, encourage the adolescents to participate in MVPA, and talk about the benefits of physical activity [21,22]. However, few studies have addressed these factors among adolescents in China, which is the most populous country in the world. Therefore, we examined the levels of MVPA among junior high school students in Xi’an, a city in Northwest China, and explored several factors that may be associated with IPA.

## 2. Materials and Methods

### 2.1. Ethics Statement

The study was approved by the Biomedical Ethics Committee of the Department of Medicine Xi’an Jiaotong University (Project identification code: 2014-596). Prior to data collection, all students and their parents had received informed consents of the present study and were assured that they would participate in this study anonymously. Participants were also informed that they could refuse to participate or to answer any questions and could withdraw at any time.

### 2.2. Study Sample

We randomly selected 9 junior high schools from the 240 schools in Xi’an using a random number table. Then, three classes of different grades per school were selected through drawing lots. All students in the chosen classes (1105 students) were invited to participate in this study, but 45 individuals refused to participate. Consequently, 1060 students aged 12 to 15 years participated in this study. And 28 subjects were excluded due to missing data, leaving 1032 students for analysis.

### 2.3. Measurement of Demographic Characteristics

Demographic information included the students’ sex, age, home address, SES, weight, and height. The weight and height of each participant were measured twice by study personnel. A third height and/or weight measurement was taken in the rare event that the first two measurements were not in agreement. Body mass index (BMI) was derived from weight and height (kg/m^2^), and thereafter BMI z-scores were calculated based on growth reference algorithms developed by the WHO for children and youth [23]. The students were grouped into the following BMI status: underweight (BMI <15th percentile), average weight (BMI 15th to 85th percentile), overweight (BMI 85th to 95th percentile), and obese (BMI >95th percentile). SES was assessed by family annual income, which was reported by parents of the adolescents. Participants were grouped into three SES: low, median, and high (<120,000 RMB, 120,000 to 300,000, and >300,000 RMB, respectively).

### 2.4. Family Support Assessment

We used Lee’s family support for physical activity scale (6 items) to measure family support level in the present study [24]. A lower score indicates lower family support for physical activity. We classified the family support distribution according to the percentile scores, dividing the subjects into two groups: high (above the 50th) and low (below the 50th). In the present study, the Cronbach’s alpha was 0.80 and the test-retest reliability was 0.83 (*n* = 50), consistent with estimates from a previous study [24].

### 2.5. Physical Activity Assessment

Physical activity was assessed using the Physical Activity Questionnaire for Middle School Students (PAQMSS), which we modified from the International Physical Activity Questionnaire [25] to specifically assess the physical activity of Chinese middle school students. The PAQMSS was revised according to the results from a qualitative survey (*n* = 18). The reliability and criterion validity of the revised questionnaire was assessed using ActiGraph GT3X triaxial accelerometers that were worn on the right hip of the participants. The activities were classified into three groups: light, moderate, and vigorous (2–3.9 MET, 4–5.9 MET, more than 6 MET) according to the intensity of energy consumption, which was recorded by the accelerometers automatically. The correlation coefficients of validity ranged from 0.584 (long jump) to 0.684 (walking) and the Cronbach’s alpha was 0.731 (*n* = 50). Participants completed the PAQMSS in the classroom in the presence of trained graduate students, who helped the students with any questions or concerns regarding the questionnaire (e.g., whether they should convert the unit “hours” into “minutes” or not.). The PAQMSS included four domains (types, frequency, duration, and intensity of physical activity), and 25 specific physical activities including walking, running, biking, skipping, kicking shuttlecock, swimming, dancing, basketball and other physical activities. Participants were asked the frequency and duration for each activity during last week. The intensity of physical activities was assessed as “none”, “a little”, “quite a bit” and “very much”, which describe the increase in breath and heartbeat after each activity. The categories “quite a bit” and “very much” were considered moderate and vigorous physical activity, respectively. The total time of MVPA was calculated by summing the self-reported frequency and duration for the specific physical activities. IPA was defined as engaging in less than 300 min per week of moderate to vigorous physical activity [5,15].

### 2.6. Data Management and Statistical Analysis

Data management and statistical analysis were performed using Epidata (version 3.1) (The Epidata Association, Odense, Denmark) and SPSS (Statistical Product and Service Solutions) for Windows (version 21.0) (IBM, Armonk, NY, USA). Results were presented as numbers and percentage for categorical data and were expressed as mean and standard deviation for continuous data. T-tests, analysis of variance and chi-squared tests were performed to analyze differences in demographic data and other study variables. A logistic regression model was used to determine the independent factors related to IPA, with the adjustment of predictive variables including age [15], gender [5,6], BMI status [9], family support for physical activity [17,18], and SES [26]. Age, gender and family support for physical activity were used as dichotomized variables, whereas BMI status and SES were coded as ordered categorical variables in logistic regression models. Statistical significance was set at *p* < 0.05, and all tests were two-sided.

## 3. Results

### 3.1. Demographic Characteristics of Participants

The mean ages of boys and girls in our study population were 13.8 ± 0.8 and 13.9 ± 0.9 years, respectively (Table 1). Compared with girls, more boys were overweight (12.8% *vs.* 7.1%, *p* < 0.001) and obese (28.2% *vs.* 14.8%, *p* < 0.001). Overall, 30.1% of boys and 43.0% of girls did not meet the recommendation of 300 minutes MVPA per week. In contrast, boys (58.1%) showed a high level of family support for physical activity than girls (37.9%; *p* = 0.002). Boys (40.1%) showed a slightly higher prevalence of low SES than girls (35.9%; *p* < 0.001).

### 3.2. Description of Physical Activity

Girls (515 ± 497 min/week) were less active than boys (828 ± 701 min/week) and had a higher percentage of IPA (43.0% *vs.* 30.1%). Compared with underweight subjects, obese adolescents were more likely to report IPA (17.8% *vs.* 52.4%). Students from families with high SES (53.6%) also showed a higher prevalence of IPA compared with those from families with low (38.4%) and medium SES (32.1%). Students who reported high family support for physical activity were less likely to report IPA (29.0%) than those reporting low family support (43.0%). On weekdays, there was no significant association between screen time and IPA. In contrast, there was an inverse association between screen time and MVPA on weekends (Table 2).

### 3.3. Influential Factors of Insufficient Physical Activity

Multivariate analyses found that boys (OR (odds ratio) = 0.7, 95% CI (confidence interval): 0.5–0.9), adolescents with underweight status (OR = 0.5, 95% CI: 0.3–0.7), and those with perceived high family support for physical activity (OR = 0.7, 95% CI: 0.6–0.9) were less likely to report IPA (Table 3). In contrast, those who were obese (OR = 2.2, 95% CI: 1.5–2.9) and of high SES (OR = 2.4, 95% CI: 1.3–4.8) were more likely to report IPA.

## 4. Discussion

Our findings suggest that Chinese youth, much like youth in Western countries, suffer from IPA [3,4]. Similar to what has been found in Western countries, girls, obese adolescents, and those with low family support for physical activity were more likely to report IPA [5,6]. High SES was positively associated with IPA among junior high school students, which has not been shown consistently in previous studies [20,27].

The present study is largely consistent with previous studies in China showing a high prevalence of IPA, although our data suggest a slight improvement over time [5,6]. In order to promote physical activity among students, the Chinese government had issued a series of guidelines since 2007 [28], requiring primary and middle schools to provide at least two physical education sessions per week, including an additional 60 min of daily exercise after class. Although still unclear, these policies may help to explain why physical activity appears to have increased among Chinese youth in the past few years.

Family support may influence physical activity [17,29,30]. Vander *et al.* suggested that increased parental encouragement was positively associated with boys’ and girls’ physical activity on school days and girls’ physical activity on weekends [30]. A systemic review of studies published between 1998 and 2013 indicated the important aspects of parenting in children’s physical activity, including parent encouragement and support [29]. This conclusion seems reasonable considering that parents often provide their children transportation, equipment and time for doing physical activity [17]. Parents’ participation in the activity also appears to increase physical activity in children [31,32]. Thus, physical activity interventions with adolescents should consider increasing family support level, while increased family support for physical activity is helpful to motivate MVPA and reduce the risk of IPA.

Our study found that boys engaged in more MVPA than girls, a finding consistent with a recent study in Beijing, China [6]. Higher physical activity in boys has also observed in Kenya [10], Canada [33], and Germany [34]. Explanations for this trend include societal roles that emphasize sports and outdoor activities for boys [5,35]. Boys may also biologically be more inclined to engage in moderate to vigorous physical activity [11]. The results of the present study also suggest that boys have higher levels of family support than girls, which may also contribute to the gender disparity in physical activity. This finding further highlights the importance of increasing family support for physical activity.

We observed an inverse association between BMI and physical activity, a finding consistent with those of other study [23,36]. For example, studies by both Biernat [19] and Borges [36] reported that having a higher BMI significantly reduced the probability of meeting recommended MVPA. However, in most studies it is not clear whether IPA resulted in obesity and overweight, or whether obesity contributed to IPA. One might expect that IPA may result in a vicious cycle, whereby increasing BMI promotes further sedentary behavior.

Our study suggests that high SES is inversely correlated with physical activity, which is not consistent with previous studies in Western countries [23,27]. Lopes *et al.* reported that high income increased the risk of IPA [37]. On the contrary, it was found that the lower income neighborhoods had lower physical activity compared with middle and higher income neighborhoods [20]. In addition, Graham suggested that a low SES was associated with lower physical activity [38]. Although it is not currently clear, perhaps our finding of an inverse association between physical activity and SES may be due to the fact that children living in a family with no cars need to walk more and therefore have a higher level of physical activity [26].

Studies of the correlation between age and physical activity have shown mixed results. One study showed that students aged 17 to 19 had a higher risk of IPA than those aged 13 to 16 years [15]. Another study stated that a significant age-gradient in adults’ physical activity could be seen in China, Ghana, Mexico, India, Russia and South Africa, where the prevalence IPA also consistently increased with increasing age [39]. Bastos reported the prevalence of IPA increased with age in girls but not in boys [31]. In contrast, no significant association between age and the level of physical activity was found in the present study. The sample age ranged from 12 years to 15 years old, so this null finding may be due to the relative narrow age range in our sample.

Several limitations of our study must be considered. We collected data of physical activity in weekly increments. Therefore, daily patterns in physical activity were not considered. Also, data were collected in spring and summer, during which higher physical activity is to be expected [40]. Further, the present study was limited by the self-reported exposure to physical activity, which may be less reliable than standardized accelerometer measurements. However, the PAQMSS was shown to be a reliable and reasonably valid instrument through pretest *via* ActiGraph GT3X triaxial accelerometers in a small sample (*n* = 50). Lastly, the present study was conducted in only one city, and therefore may not be generalizable to the entire country. However, Xi’an is considered a typical city in Northwest China, therefore, may be representative of a considerable number of communities in China.

## 5. Conclusions

In summary, this study estimated the prevalence of IPA among junior high school students in Xi’an City, China. IPA was positively associated with female gender, obesity, high family income, and low family support level. These findings may be useful in the implementation of targeted interventions to prevent the many known health consequences of a sedentary lifestyle.

## Figures and Tables

**Table 1:** Distribution of demographic data among junior high school students (*n* = 1032).

Variables	Boys (*n* = 539)	Girls (*n* = 493)	*t*	*X^2^*	*p*
	*n* (%)	*n* (%)			
Age (Mean ± SD)	13.8 ± 0.8	13.9 ± 0.9	−1.880		0.078
BMI status				45.596	<0.001
Average weight	248 (46.0)	281 (57.0)			
Underweight	70 (13.0)	104 (21.1)			
Overweight	69 (12.8)	35 (7.1)			
Obese	152 (28.2)	73 (14.8)			
Socialeconomic status				27.389	<0.001
Low	216 (40.1)	177 (35.9)			
Medium	259 (48.1)	296 (60.0)			
High	64 (11.8)	20 (4.1)			
Family support level				10.053	0.002
Low	226 (41.9)	306 (62.1)			
High	313 (58.1)	187 (37.9)			
Time to walk from home to recreation/parks				3.835	0.147
<10 min	153 (28.4)	120 (24.3)			
10–20 min	228 (42.3)	203 (41.2)			
>30 min	158 (29.3)	170 (34.5)			
Time spent outside				0.157	0.925
<1 h	301 (37.3)	182 (36.9)			
1–2 h	299 (55.5)	278 (56.4)			
>2 h	39 (7.2)	33 (6.7)			
Screen time					
Week days				0.026	0.873
≤2 h	384 (71.2)	349 (70.8)			
>2 h	155 (28.8)	144 (29.2)			
Weekend days				0.004	0.947
≤2 h	199 (36.9)	183 (37.1)			
>2 h	340 (63.1)	310 (62.9)			
Insufficient physical activity				18.667	<0.001
Yes	162 (30.1)	212 (43.0)			
No	377 (69.9)	281 (57.0)			

Notes: SD: standard deviation; BMI: body mass index.

**Table 2:** Comparison of the amounts of moderate to vigorous physical activity (MVPA) and insufficient physical activity (IPA) (*n* = 1032).

Variables	MVPA			*t*	*F*	IPA *n*/Total (%)	X^2^
	(Min/Week)	Median	25th–75th Percentile				
Gender				6.699 **			18.677 **
Boys	828 ± 701	610	290–1215			162/539 (30.1)	
Girls	515 ± 497	390	205–775			212/493 (43.0)	
BMI status					7.620		51.455 **
Average weight	736 ± 603	470	230–860			190/529 (35.9)	
Underweight	655 ± 661	591	327–1088			31/174 (17.8)	
Overweight	812 ± 694	566	260–1005			35/104 (33.7)	
Obese	547 ± 514	619	302–1297			118/225 (52.4)	
SES					9.138		15.902 **
Low	724 ± 568	655	317–992			151/393 (38.4)	
Medium	685 ± 59	502	278–1037			178/555 (32.1)	
High	520 ± 607	413	215–915			45/84 (53.6)	
Family support level				−4.54 **			22.01 **
Low	616 ± 602	391	155–703			229/532 (43.0)	
High	855 ± 747	702	382–1312			145/500 (29.0)	
Time to walk from home to recreation					13.366		0.092
<10 min	751 ± 596	671	321–987			101/273 (37.0)	
10–30 min	699 ± 604	515	298–1015			155/431 (36.0)	
>30 min	642 ± 621	443	233–998			118/328 (36.0)	
Time spent outside					52.229 **		2.102
<1 h	445 ± 569	492	229–722			128/383 (26.5)	
1–2 h	731 ± 711	687	362–969			219/577 (38.0)	
>2 h	829 ± 738	722	325–947			27/72 (37.5)	
Screen time							
Week days				−1.666			0.585
≤2 h	674 ± 638	599	313–1049			271/733 (37.0)	
>2 h	743 ± 589	697	390–1003			103/299 (34.4)	
Weekend days				5.147 **			2.353
≤2 h	832 ± 721	701	388–1309			127/382 (33.2)	
>2 h	607 ± 598	391	187–883			247/650 (38.0)	

Notes: ** *p* < 0.01; MVPA: moderate and vigorous physical activity; IPA: insufficient physical activity; BMI: body mass index; SES: socioeconomic status.

**Table 3:** Influential factors of insufficient physical activity among junior high school students (*n* = 1032).

Variables	Crude OR	95% CI	*p*	Adjusted OR	95% CI	*p*
Age						
12–13 (ref)	1					
14–15	1.2	0.7–2.4	0.57			
Gender						
Girls (ref)	1			1		
Boys	0.6	0.4–0.7	0.000	0.7	0.5–0.9	0.01
BMI status						
Average weight (ref)	1			1		
Underweight	0.4	0.3–0.6	0.000	0.5	0.3–0.7	0.004
Overweight	0.9	0.6–1.4	0.66	1.0	0.7–1.7	0.72
Obese	2.0	1.4–2.7	0.000	2.2	1.5–2.9	0.000
Socioeconomic status						
Low (ref)	1			1		
Medium	0.8	0.6–1.0	0.04	0.8	0.6–1.0	0.25
High	1.9	1.2–3.0	0.01	2.4	1.3–4.8	0.007
Family support level						
Low (ref)	1			1		
High	0.5	0.4–0.7	0.000	0.7	0.6–0.9	0.004

Notes: OR: odds ratio; CI: confidence interval; BMI: body mass index.

## Abbreviations

The following abbreviations are used in this manuscript:

MVPA	Moderate and Vigorous Physical Activity
IPA	Insufficient Physical Activity
OR	Odds Ratio
CI	Confidence Interval
SES	Socioeconomic Status
WHO	World Health Organization
PAQMSS	Physical Activity Questionnaire for Middle School Students
BMI	Body Mass Index
kg/m^2^	Kilogram per Square Meter
RMB	Renminbi
SPSS	Statistical Product and Service Solutions
